# Septic Abortion Secondary to Peptoniphilus asaccharolyticus Complicated by Bacteremia: A Case Report and Review of Literature

**DOI:** 10.7759/cureus.35978

**Published:** 2023-03-10

**Authors:** Abdulhakeem Althaqafi, Adeeb Munshi, Hadeel Altayib, Nawaf Alsubhi, Dheyyaa Alnajar, Abdulfattah Al-Amri

**Affiliations:** 1 Medicine/Infectious Diseases, King Abdullah International Medical Research Center, Jeddah, SAU; 2 Infectious Diseases, King Saud Bin Abdulaziz University for Health Sciences, Jeddah, SAU; 3 Infectious Diseases, King Abdulaziz Medical City, Jeddah, SAU; 4 Infectious Disease, King Saud Bin Abdulaziz University for Health Sciences, Jeddah, SAU; 5 Microbiology, King Abdullah International Medical Research Center, Jeddah, SAU; 6 Microbiology, King Saud Bin Abdulaziz University for Health Sciences, Jeddah, SAU; 7 Microbiology, King Abdulaziz Medical City, Jeddah, SAU

**Keywords:** abortion, septic, bacteremia, asaccharolyticus, peptoniphilus

## Abstract

*Peptoniphilus* species are gram-positive anaerobic coccus (GPAC) that are found in the human flora, such as the skin, gastrointestinal tract, and genitourinary tract, and can be pathogenic. Septic abortion caused by *Peptoniphilus* species has been reported only three times in the literature. Here, we present a case of *Peptoniphilus asaccharolyticus (P. asaccharolyticus)* bacteremia as a complication of septic abortion.

## Introduction

*Peptoniphilus* species are gram-positive anaerobic coccus (GPAC) that are usually found as part of the human flora and have the potential to become pathogenic in the presence of certain factors [1,2]. These species are found in the skin, gastrointestinal tract, and genitourinary tract [3]. *Peptoniphilusa asaccharolyticus (P. asaccharolyticus)* is the most common cause of chronic wound infections and diabetic foot infections among all *Peptoniphilus* species [4].

Septic abortion can happen after a spontaneous or induced abortion, such as medical or surgical pregnancy termination, usually caused by an upper genital tract infection [5]. However, its course is benign, particularly in places where medical care is advanced and available. Rapid recognition of the infection, broad-spectrum antibiotics and intravenous (IV) fluids, and removal of infected intrauterine tissue are the corners in managing a septic abortion [6]. Here, we report a case of *P. asaccharolyticus* bacteremia as a complication of septic abortion.

## Case presentation

A 36-year-old female, known case of asthma on inhalers, gravida 8 para 5+2, presented to the emergency department (ED) with a one-day history of fever (39 °C) recorded at home, associated with chills and vaginal bleeding. She denied any history of neurological, respiratory, or gastrointestinal complaints. One week before this presentation, she was admitted under obstetrics and gynecology as a case of incomplete abortion. The gestational age was 14 weeks at the time of the abortion. She received oral misoprostol 600 mcg once and was discharged in stable condition.

Physical examination at ED showed blood pressure was 115/77 mmHg, heart rate 101 beats per minute, respiratory rate was 20 breaths per minute, and oxygen saturation was 100% on room air. Abdominal examination revealed a soft abdomen with mild suprapubic abdominal tenderness. A sterile speculum examination revealed blood in the vagina but no active bleeding from the cervix. Another systemic examination was not remarkable, including a cardiopulmonary examination.

She had elevated white blood cell (WBC) with neutrophilic shift and high C-reactive protein (CRP). She had a normal renal and liver profile (Table 1).

**Table 1:** Laboratory investigation of the patient upon initial presentation WBC: White blood cell; LDH: Lactate dehydrogenase; CK: Creatine kinase; BUN: Blood urea nitrogen; GGT: Glutamine aminohydrolase; AST: Aspartate aminotransferase; ALT: Alanine transaminase; ALP: Alkaline phosphatase; INR: international normalized ratio; CRP: C-reactive protein; PCR: Polymerase chain reaction

Parameter	Value	Reference range
Hemoglobin	8	13.0 – 18.0 g/dL
WBC	13	4.0 – 11.0 × 10^9^/L
Neutrophil count	9	2.0 – 7.5 × 10^9^/L
Platelet count	462	150 – 450 × 10^9^/L
Lymphocyte count	1.1	1.5 – 4.0 × 10^9^/L
Potassium	4	3.5 – 4.9 mmol/L
Sodium	137	135 – 144 mmol/L
LDH	170	100 – 217 U/L
CK	30	45 – 200 IU/L
High Sensitivity Troponin I	5	1.9 – 15.6 pg/mL
Creatinine	39	65 – 112 µmol/L
BUN	2.1	2.8 – 7.4 mmol/L
Direct Bilirubin	2.8	0 – 9 µmol/L
Total Bilirubin	8	3.4 – 22.1
Total Protein	70	66 – 83 g/L
Albumin	40	39 – 50 g/L
GGT	30	11 – 68 IU/L
AST	20	5 – 34 IU/L
ALT	22	7 – 44 U/L
ALP	44	39 – 114 U/L
INR	1	0.8 – 1.2
Procalcitonin	0.08	< 0.25 µg/L
CRP	108	0 – 5 mg/L
COVID-19 PCR	Negative	
Urinalysis		
Nitrite	Negative	
Leukocyte Esterase	Negative	

She was admitted under obstetrics and gynecology and shifted to the labor and delivery room with urgent transvaginal US, which showed retained products of conception.

**Figure 1:**
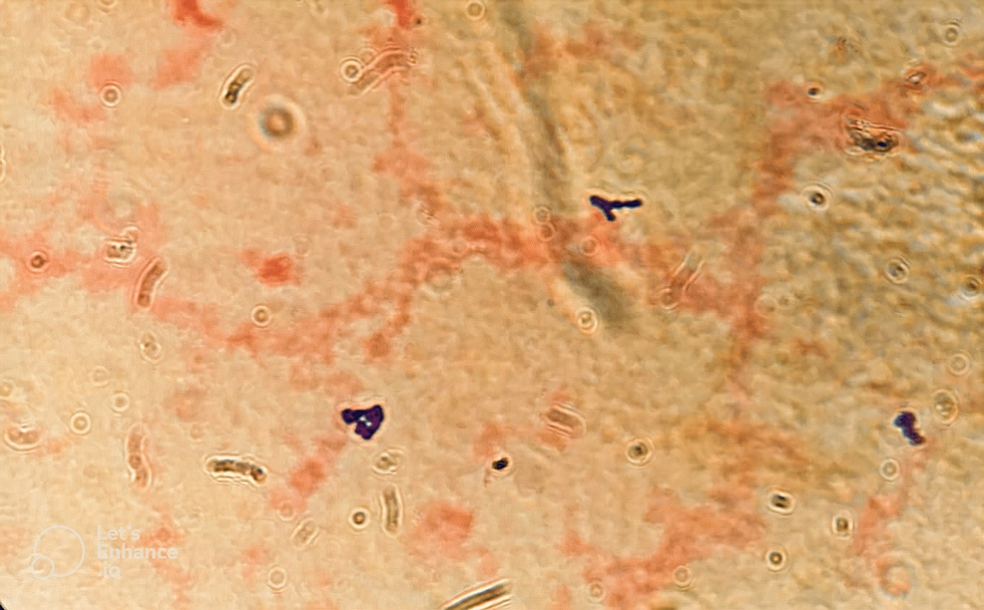
Gram stain of the blood culture showing gram-positive cocci in clusters

She underwent urgent dilation and curettage and was started on IV ampicillin 2000 mg six times per day, IV clindamycin 900 mg three times per day, and IV gentamicin 300 mg once a day. Both the dilation and curettage and the start of antibiotics brought down the fever after 24 hours. On the third day of admission, a blood culture showed *P. asaccharolyticus* identified using matrix-assisted laser desorption/ionization-time of flight (MALDI-TOF). Sensitivity of the blood culture was done using disk diffusion methods on blood agar, which showed to be sensitive to vancomycin, penicillin, and metronidazole. The gram stain smear from the culture plate showed gram-positive cocci in clusters (Figure 1).

After five days of surgical management and starting antibiotics (ampicillin, clindamycin, and gentamicin), the infectious disease (ID) team consulted and recommended stopping gentamicin and clindamycin. Additionally, the ID team recommended continuing on IV ampicillin for a total duration of seven days (two more days) and then switching the patient to oral amoxicillin/clavulanic acid 1 gram twice daily to complete a 14-day total duration of all antibiotics given. She was discharged home in stable clinical condition. She was followed up by the obstetrics and gynecology team in the clinic two weeks after discharge; she was stable without any clinical manifestations of infection. Additionally, a repeated blood culture revealed a negative result.

## Discussion

*P. asaccharolyticus*, a GPAC, belongs to the phylum Firmicutes' *Peptostreptococcaceae* family. Two *Peptoniphilus* species were initially placed in the *Peptococcus* genus, moved to the *Peptostreptococcus* genus, and then moved back to the *Peptoniphilus* genus in 2001. The name *Peptoniphilus* (peptone + philic) comes from *P. asaccharolyticus*' major metabolic byproduct, butyric acid, and its primary energy sources, peptone and amino acids. Acid is not produced using carbs. Although certain strains of *P. asaccharolyticus* generate indole, most are urease, coagulase, and alkaline phosphatase negative. This genus's cells have a diameter that varies from 0.5 to 1.5 m and form circular, opaque colonies that are 1 to 2 mm in diameter [3].

The surfaces of the human mouth, upper respiratory tract, gastrointestinal system, and genitourinary tract are colonized by a specie of bacteria known as GPAC. These commensals normally do not cause infection and disease due to naturally present protective barriers, but in some situations and populations, GPAC may behave like opportunistic pathogens and result in clinically important infections. Elderly people, those who have had prosthetic joints or other foreign objects introduced, and immunocompromised individuals are all at increased risk of GPAC becoming pathogenic. About 25% to 30% of anaerobic infections in humans are caused by GPAC. These are frequently identified by cultures taken from suffering female genital tracts and abscesses in the skin, soft tissue, mouth, bones, and joints [1].

Due to the polymicrobial nature of these infections, as well as the difficulties in culturing these bacteria, individual GPAC has not been thoroughly explored.

Anaerobe isolation requires lengthy cultivation periods and delaying results until the infection has already started on treatment. However, with the growing use of non-biochemical techniques like 16S rRNA sequencing methods and MALDI-TOF, they are more frequently being reported and correctly identified. Since 2012, seven new species of *Peptoniphilus*, among other bacterial species of therapeutic significance, have been described thanks to gene sequencing [2].

There have been many reports in the literature revealing that the *Peptoniphilus* bacteria were isolated from bone, joint, skin, and soft tissue infections as well as bloodstream infections [2,7]. Not only that, but it was also found in abscesses from different body parts like kidneys, peritonsillar, and even spinal abscesses [8-10]. A review of 15 cases of *Peptoniphilus* bloodstream infection was done in Canada from 2007 to 2011; three patients were found to have a septic abortion with chorioamnionitis [2]. The usual patients at risk for infections with these bacteria are the elderly, immunocompromised, as well as post-surgical patients [7]. Most *Peptoniphilus *infections were polymicrobial, and only minimal case reports were found in the literature describing monomicrobial infections [10].

The typical organism leading to septic abortion includes *group A streptococcus* and *Escherichia coli (E. coli)*, and less commonly cultured are bacteroids as well as *Peptostreptococcus* [11]. This is, however, the fourth case in the literature leading to a septic abortion caused by* P. asaccharolyticus*. The medical treatment for these bacteria is usually based on susceptibility. They can be responsive to penicillin, piperacillin/tazobactam, vancomycin, and tigecycline. Dalbavancin and ceftobiprole were also found to be particularly effective in the eradication of *P. asaccharolyticus* [12,13]. Resistance against clindamycin has been reported; thus, susceptibility testing of this organism is warranted. Moreover, surgical options may be warranted in certain cases such as bone and joint infections [3].

## Conclusions

In conclusion, the rare case presented in this study demonstrates that *P. asaccharolyticus* induced by host factors can cause severe monomicrobial infections. The patient underwent urgent dilation and curettage and was treated with seven days of IV ampicillin and was then discharged home on another seven days of oral amoxicillin/clavulanic acid. Clinicians should be alerted to infections caused by *Peptoniphilus* species and they should pay attention to the importance of anaerobic culture. Prompt antibiotic therapy combined with surgical treatment can effectively treat infections caused by this organism.
